# Vascularized parallel-ridge pattern: dermoscopic sign in acral melanoma with anatomopathological correlation^⋆^

**DOI:** 10.1016/j.abd.2023.09.010

**Published:** 2024-07-15

**Authors:** Elisa Scandiuzzi Maciel, Masiel Garcia Fernandez, Milvia Maria Simões e Silva Enokihara, Sérgio Henrique Hirata

**Affiliations:** a Department of Dermatology, Escola Paulista de Medicina, Universidade Federal de São Paulo, São Paulo, SP, Brazil; b Department of Pathology, Escola Paulista de Medicina, Universidade Federal de São Paulo, São Paulo, SP, Brazil

*Dear Editor,*

Acral lentiginous melanoma is a rare subtype of melanoma that affects the palms, soles and nails. Its early diagnosis is challenging, mainly due to a higher proportion of amelanotic melanomas and a wide variety of clinical presentations.^1^ Patients often present with advanced disease at the time of diagnosis and therefore have a worse prognosis when compared to other melanoma subtypes.^2^ Dermoscopy of the lesions shows specific patterns, being of great value for an early and accurate diagnosis. This is a case report of a recently described dermoscopic sign.

A 61-year-old woman reported the appearance of a brownish lesion on the right plantar region three years before, with progressive growth and ulceration. She had the lesion excised at other hospital, but the material was not sent for anatomopathological study. She was then referred to ours hospital.

On clinical examination, she had a surgical scar in good aspect, measuring approximately 3 cm, on the right plantar region. Contact dermoscopy with polarized light of the skin adjacent to the scar showed erythema and dotted vessels filling the ridges and sparing the furrows (Fig. 1) ‒ a recently described dermoscopic pattern, called “vascularized parallel-ridge pattern”.^3^

**Figure 1:**
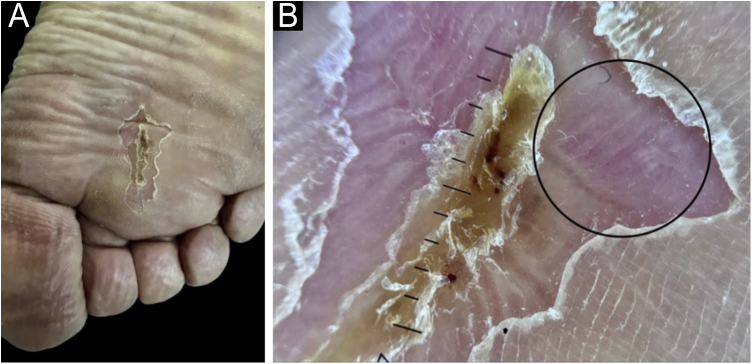
Patient's right plantar region, in the first evaluation at the hospital. On the left, the clinical photo shows the surgical scar from the previous excision at another medical service. On the right, contact dermoscopy with polarized light of the skin adjacent to the scar highlights the “vascularized parallel-ridge pattern” in the area circled in black.

The clinical history and the identification of the vascularized parallel-ridge pattern led to a high suspicion of melanoma. Therefore, a wider ressection with a 2-cm margin was performed. Histopathology showed residual melanoma in the deep dermis (Figure 2, Figure 3) and surgical margins free of neoplasia. In correspondence with the dermoscopic changes there were grouped proliferated and dilated capillary vessels close to the eccrine ducts (Fig. 4).

**Figure 2:**
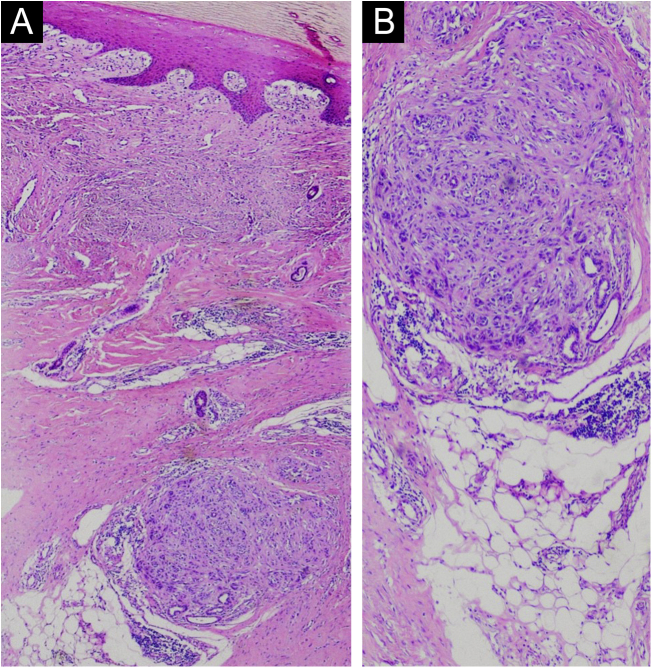
In the deep dermis, a circunscribed area of atypical morphology suggesting melanoma (likely residual or recurrent) (Hematoxylin & eosin, ×40 on the left and ×100 on the right).

**Figure 3:**
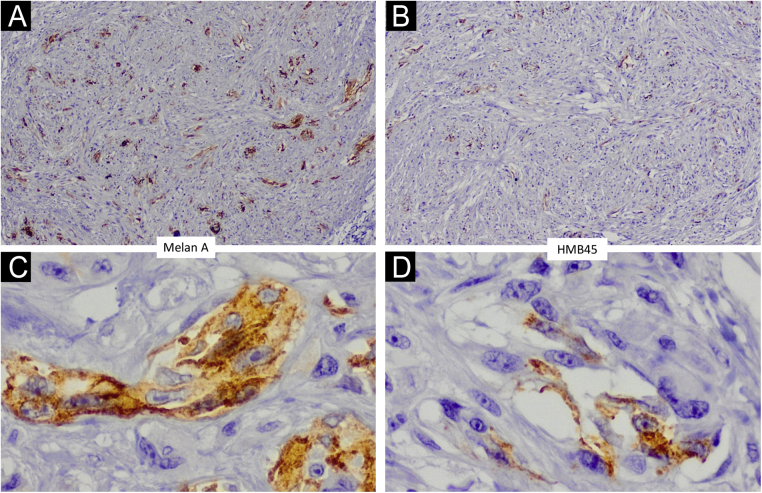
Immunohistochemistry confirmed the presence of atypical Melan A and HMB45 positive melanocytic cells (×40 in the upper photos and ×400 in the lower photos).

**Figure 4:**
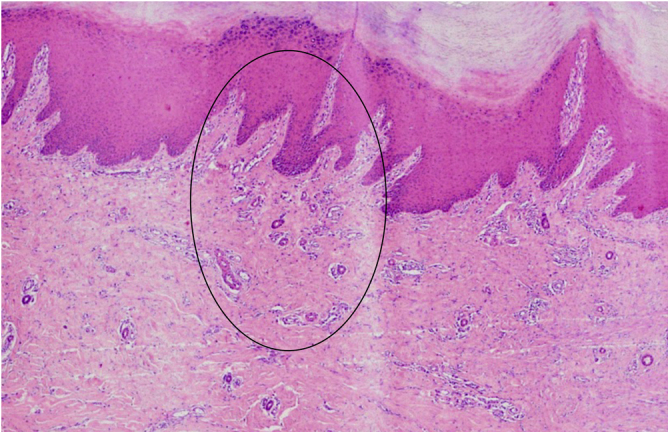
Grouped proliferated dilated capillary vessels close to the eccrine ducts, in the area circled in black (Hematoxylin & eosin ×40).

Acral lentiginous melanomas can show characteristic dermoscopic patterns of great diagnostic value. For pigmented lesions, three patterns should be highlighted: the parallel-ridge pattern with a sensitivity of 86% and specificity of 99%; the irregular diffuse pigmentation, which seems to be more suggestive of invasive acral lentiginous melanomas; and the multicomponent pattern, characterized by the presence of three or more dermoscopic patterns in the same lesion.^1^, ^4^, ^5^ For amelanotic lesions, which correspond to up to 34% of acral lentiginous melanomas, atypical or polymorphic vascular patterns stand out.^1^ The multicomponent pattern and the atypical or polymorphic vascular patterns are not specific to volar skin, since they are also found in melanomas located in other regions.^1^

In 2018, Ozdemir et al. described a new dermoscopic sign for acral lentiginous melanoma, called “vascularized parallel-ridge pattern”, characterized by erythema and dotted vessels filling the ridges and sparing the furrows. In that population (n = 46), the prevalence of this sign was 13.6% among pigmented lesions and 36.4% among amelanotic melanomas.

Histopathology of early acral lentiginous melanomas suggests that the proliferation of atypical melanocytes occurs preferentially in the crista intermedia - which corresponds on dermoscopy to the parallel-ridge pattern.^6^ Ozdemir et al. postulate that vascularization and inflammation are more intense in places where there is a greater proliferation of atypical melanocytes, that is, in the crista intermedia, which would manifest itself on dermoscopy as the vascularized parallel-ridge pattern p - especially in lesions with less pigmentation or amelanotic, where there is less pigment interference in the observation of the vascularization.^3^ The histopathological findings in the present report support this hypothesis, as the proliferation of capillary vessels was observed close to the crista intermedia.

Palmoplantar skin has a peculiar architecture and, therefore, acral lentiginous melanomas show characteristic dermoscopic patterns that are different from what is observed in other regions. Therefore, knowledge of the new vascular dermoscopic criteria described by Ozdemir et al. is important for identifying acral lentiginous melanomas - especially thin, hypopigmented, or amelanotic ones, for which, to date, there are no other specific dermoscopic criteria. The reported case illustrates the importance of this knowledge: in the presence of a previously excised lesion that did not show pigment (amelanotic) in the current examination, the diagnosis of residual melanoma was only possible by identifying the vascularized parallel-ridge pattern. The histopathological findings associated with this dermoscopic sign are described for the first time herein. Furthermore, this case also illustrates the need to always send surgical specimens for anatomopathological study.

## Financial support

None declared.

## Authors' contributions

Elisa Scandiuzzi Maciel: Collection, analysis and interpretation of data; intellectual participation in the propaedeutic and/or therapeutic conduct of the studied cases; drafting and editing of the manuscript or critical review of important intellectual content; critical review of the literature; approval of the final version of the manuscript.

Masiel Garcia Fernandez: Collection, analysis and interpretation of data; intellectual participation in the propaedeutic and/or therapeutic conduct of the studied cases; approval of the final version of the manuscript.

Milvia Maria Simões and Silva Enokihara: Collection, analysis and interpretation of data; drafting and editing of the manuscript or critical review of important intellectual content; effective participation in research orientation; intellectual participation in the propaedeutic and/or therapeutic conduct of the studied cases; approval of the final version of the manuscript.

Sérgio Henrique Hirata: Collection, analysis and interpretation of data; drafting and editing of the manuscript or critical review of important intellectual content; effective participation in research orientation; intellectual participation in the propaedeutic and/or therapeutic conduct of the studied cases; approval of the final version of the manuscript.

## Conflicts of interest

None declared.
